# Endoscopic resection of gastric cancer involving pylorus and duodenum using novel anchor ring-shaped thread counter traction

**DOI:** 10.1055/a-2641-2253

**Published:** 2025-07-25

**Authors:** Hirohito Mori, Yasunori Yamamoto, Kazuki Niida, Masaaki Tange, Yoichi Hiasa

**Affiliations:** 1 Department of Advanced and Innovative Endoscopy, Ehime University Graduate School of Medicine, Toon, Ehime, Japan; 2 Department of Gastroenterology and Metabology, Ehime University Graduate School of Medicine, Toon, Ehime, Japan

As endoscopic submucosal dissection (ESD) is an established treatment for gastric cancer, it is sometimes very difficult to dissect the lesion depending on its location. Particularly, an expanding lesion from the gastric pylorus into the duodenal bulb is very difficult to perform ESD with a high risk of duodenal perforation 1 . While various traction methods have been reported 2 3 4 5 , we report a novel traction technique without interference with the endoscope.

An 81-year-old man underwent ESD for gastric cancer. The lesion was located on the posterior wall of the pylorus with the proximal part (around 5 mm in diameter) ( Fig. 1 **a–c** ), and most of the lesion with the distal part (15 mm in diameter) was expanded into the posterior wall of the duodenal bulb ( Video 1 ). All of the lesion was revealed by pushing up the posterior wall of the pyloric ring using an endoscopic attachment ( Fig. 1 **d** ). Under retroflex view within the duodenal bulb, duodenal ESD was conducted ( Fig. 1 **e, f** ). Although submucosal dissection was performed from the duodenal side, the lesion shifted into the duodenum, making it more difficult to dissect over the pyloric ring ( Fig. 2 **a, b** ). A Zeoclip (Zeon Co., Tokyo) with a 5-mm ring-shaped thread was put on the anterior wall of the antrum, opposite side of the lesion, to be used as an anchoring clip ( Fig. 2 **c, d** ). After another Zeoclip was used to hook the ring thread, the anchor ring-thread clip was pulled to the proximal side of the resected specimen without insufflation ( Fig. 2 **e** ). With insufflation, the lesion was shifted into the stomach. Submucosal dissection was safely completed under sufficient view by counter-traction ( Fig. 2 **f** ).

**Fig. 1:**
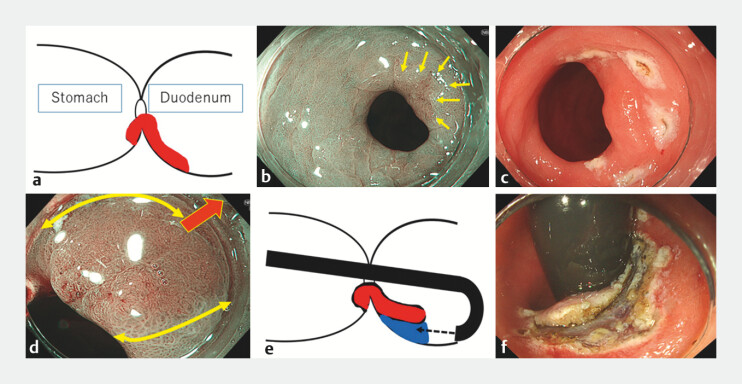
Endoscopic resection of gastric cancer involving pylorus and duodenum by anchor ring-shaped thread counter traction under retroflex view within duodenal bulb. **a–c** The lesion was located on the posterior wall of the pylorus, with a portion (approximately 5 mm in diameter) involving the posterior wall of the pyloric ring (yellow arrows). The majority of the lesion (around 15 mm in diameter) was observed extending into the posterior wall of the duodenal bulb. **d** By pressing the posterior wall outward with the endoscope attachment (red bold arrow), the majority of the lesion (around 15 mm in diameter) was observed extending into the posterior wall of the duodenal bulb (yellow curved arrows). **e, f** Under retroflex view within the duodenal bulb, submucosal dissection was conducted.

**Fig. 2:**
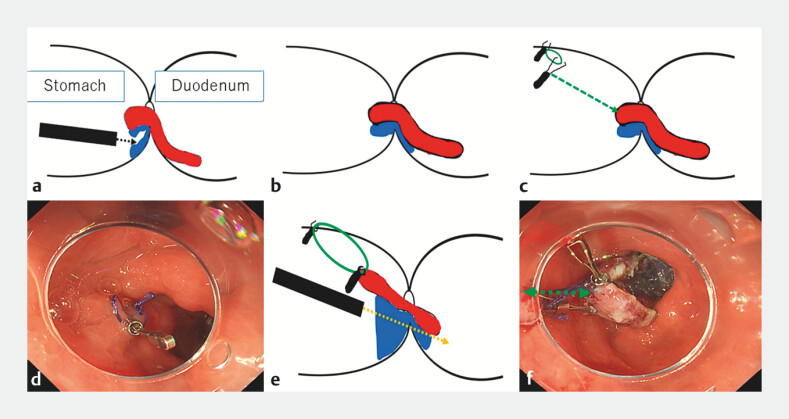
ESD of the gastric side was performed in the straightforward view using the anchor ring-shaped thread counter traction method. **a, b** After ESD of the duodenal lesion, ESD of the gastric side was performed in the straightforward view. **c, d** A Zeoclip with 5 mm ring-shaped thread was put on the anterior wall of the antrum, opposite side of the lesion, to be used as an anchoring clip. **e** After another Zeoclip was used to hook the ring thread, the anchor ring-thread clip was pulled to the proximal side of the resection specimen without insufflation. **f** With insufflation, the blind lesion on the posterior wall of the duodenal bulb pulled by the ring-thread was shifted into the gastric side (green arrow), allowing the remaining duodenal ESD to be easier within the stomach.

Novel anchor ring-shaped thread counter traction for obtaining a direct view of the gastric cancer hidden behind the pylorus.Video 1

The anchor ring-shaped thread counter-traction method was useful to secure the operative field when treating lesions expanding from the pylorus to the duodenal bulb. This technique was also useful for colorectal ESD by obtaining a direct view of lesions hidden behind mucosal folds.

Endoscopy_UCTN_Code_TTT_1AO_2AG_3AD

## Notes

Endoscopy E-Videos https://eref.thieme.de/e-videos
*E-Videos* is an open access online section of the journal *Endoscopy* , reporting on interesting cases and new techniques in gastroenterological endoscopy. All papers include a high-quality video and are published with a Creative Commons CC-BY license. Endoscopy E-Videos qualify for HINARI discounts and waivers and eligibility is automatically checked during the submission process. We grant 100% waivers to articles whose corresponding authors are based in Group A countries and 50% waivers to those who are based in Group B countries as classified by Research4Life (see: https://www.research4life.org/access/eligibility/ ). This section has its own submission website at https://mc.manuscriptcentral.com/e-videos .
